# Rapid Increases in Forest Understory Diversity and Productivity following a Mountain Pine Beetle (*Dendroctonus ponderosae*) Outbreak in Pine Forests

**DOI:** 10.1371/journal.pone.0124691

**Published:** 2015-04-10

**Authors:** Gregory J. Pec, Justine Karst, Alexandra N. Sywenky, Paul W. Cigan, Nadir Erbilgin, Suzanne W. Simard, James F. Cahill

**Affiliations:** 1 Department of Biological Sciences, University of Alberta, Edmonton, Alberta, Canada; 2 Department of Renewable Resources, University of Alberta, Edmonton, Alberta, Canada; 3 Department of Forest and Conservation Sciences, University of British Columbia, Vancouver, British Columbia, Canada; Insititute of Applied Ecology, CHINA

## Abstract

The current unprecedented outbreak of mountain pine beetle (*Dendroctonus ponderosae*) in lodgepole pine (*Pinus contorta*) forests of western Canada has resulted in a landscape consisting of a mosaic of forest stands at different stages of mortality. Within forest stands, understory communities are the reservoir of the majority of plant species diversity and influence the composition of future forests in response to disturbance. Although changes to stand composition following beetle outbreaks are well documented, information on immediate responses of forest understory plant communities is limited. The objective of this study was to examine the effects of *D*. *ponderosae-*induced tree mortality on initial changes in diversity and productivity of understory plant communities. We established a total of 110 1-m^2^ plots across eleven mature lodgepole pine forests to measure changes in understory diversity and productivity as a function of tree mortality and below ground resource availability across multiple years. Overall, understory community diversity and productivity increased across the gradient of increased tree mortality. Richness of herbaceous perennials increased with tree mortality as well as soil moisture and nutrient levels. In contrast, the diversity of woody perennials did not change across the gradient of tree mortality. Understory vegetation, namely herbaceous perennials, showed an immediate response to improved growing conditions caused by increases in tree mortality. How this increased pulse in understory richness and productivity affects future forest trajectories in a novel system is unknown.

## Introduction

Over the past century, forests in North America have experienced increased disturbances from insect outbreaks, wildfire, and harvesting [1]. Disturbances, such as wildfire and harvesting, affect resource availability, remove dominant tree species from the landscape and can increase vegetative heterogeneity in the forest system [2,3]. Though wildfire has historically been the primary agent of sudden tree mortality in western boreal forests [2,4] insect outbreaks have increased both in frequency and spatial scale [5–7]. Of the current outbreaks, that resulting from the mountain pine beetle (*Dendroctonus ponderosae* Hopkins) has emerged as the largest recorded in western Canada [8,9]. *Dendroctonus ponderosae* plays an important role in maintaining structural diversity in forest ecosystems [10,11]. It typically attacks stressed trees [9,12] when beetle populations are low; however, under epidemic conditions mass mortality of healthy overstory trees frequently occurs [6]. Unlike wildfire and harvesting, *D*. *ponderosae* leaves the dead overstory and residual understory vegetation intact [13,14].

Though there exists information on changes to overstory structure following infestation by *D*. *ponderosae* [10,15,16], response of understory plant species has received less attention. In particular we lack information on immediate responses of understories to *D*. *ponderosae*-induced tree mortality (but see [17,18]). This information is important for two reasons. First, understory plant communities can act as a strong filter on tree seedling regeneration and future forest trajectories by modifying microclimatic conditions such as light availability and soil moisture status [1,19,20]. Understory plant species may also compete with tree seedlings for resources [21,22]. Second, compensatory responses by residual understory vegetation are likely to be an important component for predicting resources available following *D*. *ponderosae*-induced tree mortality [23]. For example, compensatory responses accounted for about half of the nitrate retained in forests by surviving residual vegetation following *D*. *ponderosae* outbreak in lodgepole pine forests in Colorado [23]. Quantification of productivity in residual vegetation will be important to mitigate changes in soil nutrients resulting from post-mountain pine beetle harvest practices [23] aimed at stimulating tree seedling regeneration [24] and decreasing nitrate release from watersheds [23].

The objective of this study was to investigate the immediate effects of tree mortality caused by *D*. *ponderosae* on: (a) the overall understory plant community and (b) herbaceous and woody perennials individually. Specifically, the following three questions were addressed: (1) Is there a relationship between aboveground understory biomass and tree mortality? (2) Can increases in tree mortality explain changes in species richness and evenness? (3) Do changes in resource availability result in increased productivity and diversity?

## Materials and Methods

### Study Area

The study area is approximately 70 km south of Grande Prairie within the Lower Foothills natural subregion of west central Alberta (54°39'N, 118°59’W; 950–1150 m) [25]. Soils are classified as Orthic Gray Luvisols that are moderately well drained over glacial till. Forests are dominated by mature, even-aged *Pinus contorta* Douglas ex Loudon interspersed with *Picea glauca* (Moench) Voss, *Abies balsamea* (L.) Mill., *Picea mariana* Mill. Britton, Sterns, & Poggenb., *Betula papyrifera* Marshall and *Populus tremuloides* Michx. in the subcanopy. We located eleven sites within a 625-km^2^ region of recent *D*. *ponderosae* activity (since 2009) that bordered provincial permanent sampling plots (see [26] for details on site selection and stand composition and [27] for details on site locations). Permits and approval for conducting the study on protected land were obtained from Alberta Environment and Sustainable Resource Development, Anina Hundsdoerfer, Forest Health Specialist. Permanent sampling plots did not involve endangered or protected species.

### Plant Survey

In May 2012, we used a 1600-m^2^ (40 m x 40 m) area at each of the eleven sites to establish ten evenly distributed 9 m x 9 m plots for a total of 110 plots (S1 Fig). In June 2012, we recorded species identity, diameter at breast height (≥ 1.37 m), and health status of all mature trees and saplings within each plot. Attack by *D*. *ponderosae* on mature trees was confirmed by the presence of pitch tubes, boring dust, exit holes, and subcortical galleries [28]. We revisited these trees in June 2013 to document current beetle-induced tree mortality. Basal area by species was calculated for each plot and tree mortality was calculated as beetle-killed *P*. *contorta* basal area over total basal area expressed as a percentage. We established a 1 m x 1 m subplot in a random cardinal direction next to the center of each 9 m x 9 m plot across all 110 plots in May 2012 (S1 Fig). During three sampling periods (June, July, August) in 2012 and 2013, we identified individual herbaceous and woody perennials by species within all subplots (see S1 Table for detailed list). To determine biomass, we harvested all aboveground parts of the understory vegetation by species from each subplot in August 2012 and 2013. Harvested plants were dried at 70°C for 48 hours, weighed, and pooled per individual subplot. To account for any direct effects of the 2012 harvest on understory productivity and sampling in 2013, a 1 m x 1 m subplot was established and sampled adjacent to the original subplot in May 2013.

During the three sampling periods, in 2012 and 2013, all subplots were assessed for resources likely limiting plant growth in these forests, i.e., light, water and nutrients [29,30]. Light transmission readings were taken within two hours of solar noon on overcast days using a linear photosynthetic active radiation (PAR) sensor (Decagon Devices Inc., Pullman, WA, USA) above the shrub layer (~1 m). Light transmission was recorded as the ratio of PAR above the shrub layer (*L*_1_) compared with PAR above the forest canopy (*L*_2_) transformed into % PAR by the following equation: % PAR = (*L*_1_/ *L*_2_) x 100. Using a Theta Probe soil moisture sensor (Delta-T Devices, Cambridge, UK), volumetric soil moisture content was measured at the four cardinal directions in each subplot from the upper 10 cm of the soil column and pooled. Soils were sampled for macronutrients using Plant Root Simulator (PRS) probes (Western Ag Innovations, Inc., SK, Canada). In June 2012 and 2013, PRS probes were inserted 10 cm into mineral soils of the A-horizon at a sampling intensity of four cation and anion probe pairs per subplot. PRS probes were removed from the soil in August 2012 and 2013, cleaned, and shipped to Western Ag Laboratories for macronutrient analysis. Soil nitrogen concentrations were determined using an autoanalyzer, while potassium, calcium, magnesium, phosphorus, and sulfur concentrations of soils were measured by inductive-coupled plasma spectrometry.

Air temperature and relative humidity at the forest floor were measured across all sites and precipitation across the study area was taken during the three sampling periods in 2012 and 2013. In May 2012, air temperature and relative humidity sensors (HOBO U23 Pro V2 Temperature/Relative, Onset Computer Corp., Bourne, MA, USA) were housed in PVC pipe and mounted to the forest floor across seven random locations at a minimum distance of 7 m apart within each of our eleven sites. Readings were taken at thirty minute intervals from June through August 2012 and 2013. Precipitation data was taken at daily intervals for June through August 2012 and 2013; data was obtained from the Pinto Lookout meteorological station (54°78'N, 119°39’W) located within ~30 km of field sites in west central Alberta. Data was provided by the Alberta Agriculture and Rural Development, AgroClimatic Information Service (ACIS) http://agriculture.alberta.ca/acis/ (data retrieved, May 2014). Data from this study are made available at Dryad (doi: 10.5061/dryad.g23f6).

### Data Analysis

Understory species richness was calculated at the community and functional group level for each subplot. At the functional group level, we categorized species according to growth form (herbaceous and woody perennials) and calculated species richness as the number of species in a functional group. Evenness was also calculated at both the community and functional group level as: *J = H’/*log*(S)*, where *H’* is the Shannon diversity index and *S* is the total number of species. Individual species biomass was used to determine the Shannon diversity index.

We used linear mixed effects models to test for variation in productivity, species richness, and evenness across the gradient of *D*. *ponderosae*-induced tree mortality at both the community and functional group level for each year separately. Linear mixed effects models were also used to determine if variation in resource availability were associated with increased productivity and diversity of understory vegetation at both the community and functional group level for each year separately. Tree mortality, light, soil moisture and nutrients were included as fixed factors. Site was used as a random factor to account for potential spatial autocorrelation due to the clustering of subplots into sites. Since macronutrients were highly correlated to one another (*r* = 0.92), a principal components analysis (PCA) using a variance-covariance matrix [31] was conducted on the following macronutrients: nitrogen, potassium, calcium, magnesium, phosphorus, and sulfur. PCA axis 1 scores were used for nutrients on all subsequent analyses. All variables included had low levels of colinearity (i.e. *r* < 0.50). All models were analyzed using the R-package *nlme* [32]. All model assumptions were checked by visual inspection of residual patterns [33].

Using these linear mixed effects models, we developed candidate models that included all combinations of the explanatory variables and their interactions (i.e., *D*. *ponderosae*-induced tree mortality, soil moisture, light, and nutrients) and used information-criteria to rank the relative importance of those variables in the models. We used the dredge function in the R-package *MuMln* [34] for model selection, recording and ranking all statistical models using Akaike’s Information Criterion corrected for small sample sizes (AIC_C_). We also calculated an Akaike weight (*w*_i_) for each model [35]. We then defined a 95% confidence set of models for inference, summing *w*_i_ from best to worst model until the sum of *w*_i_ exceeded 95%. Models not meeting a 95% confidence set were excluded. When there was no clear parsimonious model (*w*_i_<0.90), we used the model.avg function in the R-package *MuMln* [34] for model averaging to determine the direction and magnitude of the effect of each explanatory variable (i.e., *D*. *ponderosae*-induced tree mortality, soil moisture, light, and nutrients) [35]. Instead of relying on the estimates of the best candidate model, we computed a weighted average of the estimate for a given parameter based on the Akaike weights. We further calculated the unconditional standard error (or precision; SE not restricted to the single “best” model) of the model-averaged estimate. We also computed the relative importance of the fixed factors by summing the *w*_i_ of the models that contained each factor. When an explanatory variable was not strongly ranked (Σ*w*_i_<0.50), we considered it important when the associated 95% confidence interval of the model-average estimate did not overlap with zero (i.e., there was an effect). All data analyses were run using R 3.0.1 [36].

## Results

All environmental conditions varied between years. Air temperature at the forest floor in 2012 (mean = 14.07 ± 0.009°C (SE)) was higher than in 2013 (12.99 ± 0.009°C) (*t* = 83.64, *P*<0.0001). Relative humidity measured at the forest floor was lower in 2012 (84.6 ± 0.03%) than in 2013 (90.92 ± 0.02%) (*t* = 159.3, *P*<0.0001). Precipitation across the study area was greater in 2013 (2.64 ± 0.32 mm) than in 2012 (1.84 ± 0.44 mm) (GLM; *z* = 3.01, *P* = 0.003). These results show that 2012 was a warmer and drier year than 2013.

**Fig 1:**
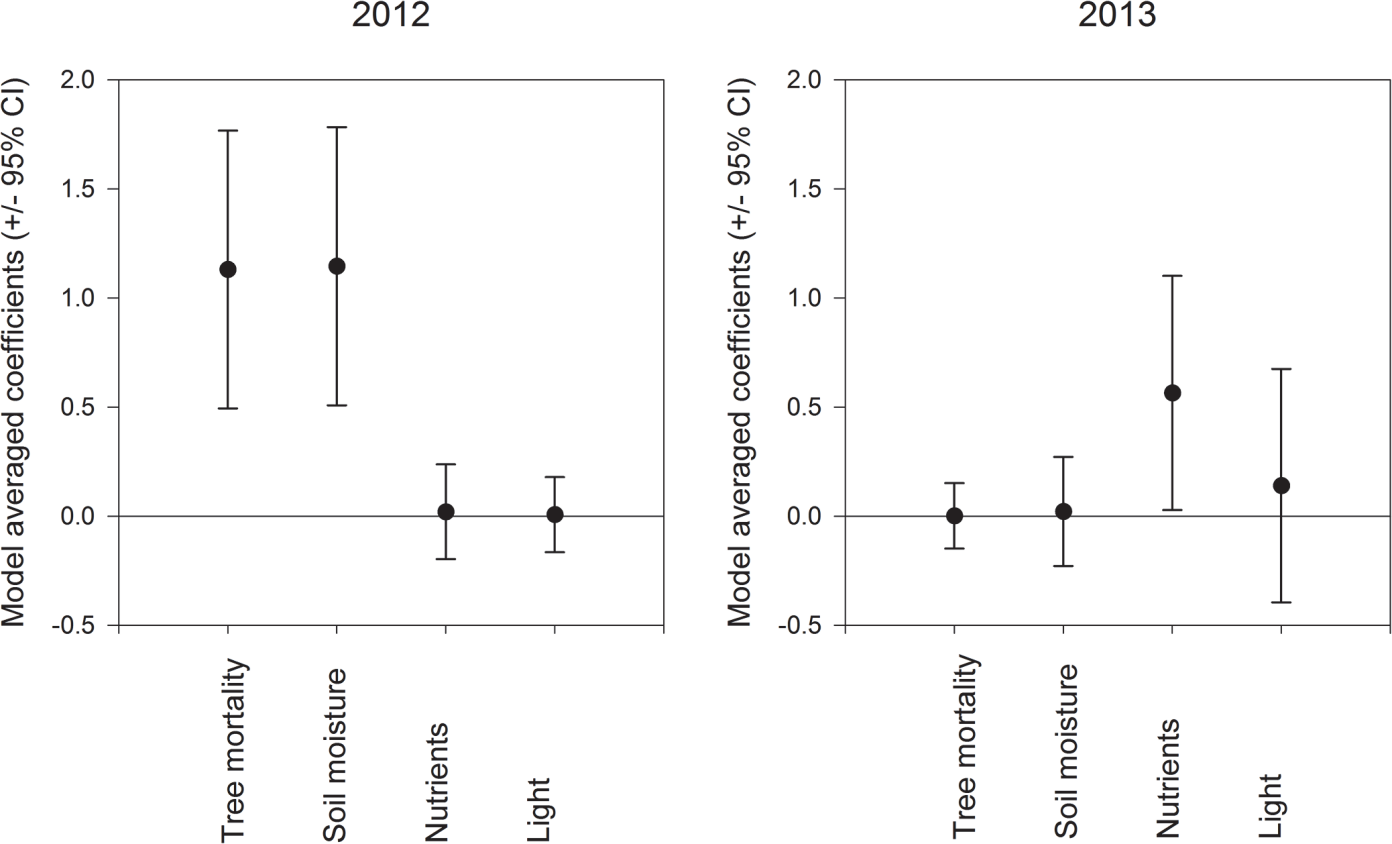
Model-averaged coefficients and 95% confidence intervals of *Dendroctonus ponderosae*-induced tree mortality, percent soil moisture, soil nutrients and light predicting understory plant community biomass in 2012 and 2013.

Model selection resulted in two models for 2012 and four models for 2013 used for inference on the factors underlying variation in total aboveground biomass (Table 1). Soil moisture and tree mortality were found to be the most important in explaining total aboveground biomass of the understory plants in 2012 (Tables 1 and 2; Fig 1). Total aboveground biomass increased with *D*. *ponderosae*-induced tree mortality and greater soil moisture levels in 2012, but not in 2013 (S2 Fig). The ranked relative importance was low for tree mortality (Σ*w*_i_ = 0.07) and soil moisture (Σ*w*_i_ = 0.17) with model-averaged coefficients for tree mortality and soil moisture also being small and uncertain in explaining total aboveground biomass in 2013 (Table 2; Fig 1). The effects of soil nutrients were found to be a strong predictor of total aboveground biomass only in 2013 (Table 2; Fig 1; S2 Fig). Ranked relative importance for light levels above the understory (but below the canopy) in explaining understory productivity along the *D*. *ponderosae*-induced tree mortality gradient were low in 2012 (Σ*w*_i_ = 0.08) and 2013 (Σ*w*_i_ = 0.26) with the model-averaged coefficient being small and showing no effect on understory productivity (Tables 1 and 2; Fig 1).

**Table 1:** Candidate models used for inference on the productivity, richness and evenness of understory plant community responses to *Dendroctonus ponderosae*-induced tree mortality, light, soil moisture, and nutrients.

**Productivity in 2012**					
	Candidate models	AIC_C_	ΔAIC_C_	*w*_i_	Evidence ratio
1	Soil moisture	478.2	0.00	0.56	1.00
2	Tree mortality, soil moisture	478.6	0.48	0.44	1.27
**Productivity in 2013**					
	Candidate models	AIC_C_	ΔAIC_C_	*w*_i_	Evidence ratio
1	Nutrients	546.3	0.00	0.40	1.00
2	Light	546.9	0.62	0.29	1.37
3	Null model (intercept only)	547.8	1.57	0.18	2.22
4	Soil moisture, nutrients, light	549.7	3.43	0.07	5.71
**Richness in 2012**					
	Candidate models	AIC_C_	ΔAIC_C_	*w*_i_	Evidence ratio
1	Soil moisture, nutrients	443.7	0.00	0.35	1.00
2	Nutrients	444.4	0.66	0.25	1.40
3	Soil moisture	445.5	1.79	0.14	2.50
4	Light	445.6	1.87	0.14	2.50
5	Tree mortality, soil moisture, nutrients	446.8	3.01	0.08	4.37
6	Null model (intercept only)	447.6	3.85	0.05	7.00
**Richness in 2013**					
	Candidate models	AIC_C_	ΔAIC_C_	*w*_i_	Evidence ratio
1	**Tree mortality, soil moisture, nutrients**	514.6	0.00	0.94	1.00
2	Soil moisture, nutrients	520.3	5.66	0.06	15.6
**Evenness in 2012**					
	Candidate models	AIC_C_	ΔAIC_C_	*w*_i_	Evidence ratio
1	**Null model (intercept only)**	-122.1	0.00	0.95	1.00
2	Tree mortality	-116.1	6.03	0.05	19.0
**Evenness in 2013**					
	Candidate models	AIC_C_	ΔAIC_C_	*w*_i_	Evidence ratio
1	**Null model (intercept only)**	-166.2	0.00	0.95	1.00
2	Tree mortality	-159.6	6.61	0.03	31.6
3	Nutrients	-157.0	9.13	0.01	95.0
4	Soil moisture	-156.7	9.44	0.01	95.0

*Notes*: The most likely models (*w*_i_>0.90; difference in evidence ratio>2.7) are shown in **bold**. AIC_C_ = Akaike’s Information Criterion corrected, ΔAIC_C_ = difference between AIC_Ci_ and AIC_C best model_, *w*_i_ = Akaike weight, and evidence ratio = *w*_j best model_ / *w*_i_.

**Table 2:** Ranked relative importance of variables associated with the productivity, richness, and evenness of the understory plant community showing model estimates of slope and variance.

**Productivity**							
2012				2013			
Explanatory variable	Σ*w*_i_	Model-average estimate	Unconditional SE	Explanatory variable	Σ*w*_i_	Model-average estimate	Unconditional SE
**Soil moisture**	0.99	1.146	0.326	**Nutrients**	0.47	0.565	0.274
**Tree mortality**	0.44	1.131	0.325	Light	0.26	0.140	0.273
Nutrients	0.08	0.020	0.111	Soil moisture	0.17	0.021	0.128
Light	0.08	0.007	0.088	Tree mortality	0.07	0.001	0.077
**Richness**							
2012				2013			
Explanatory variable	Σ*w*_i_	Model-average estimate	Unconditional SE	Explanatory variable	Σ*w*_i_	Model-average estimate	Unconditional SE
**Nutrients**	0.67	0.580	0.252	**Soil Moisture**	1.00	0.567	0.247
**Soil moisture**	0.56	0.498	0.254	**Nutrients**	1.00	0.937	0.234
Light	0.14	0.077	0.216	**Tree mortality**	0.94	0.716	0.237
Tree mortality	0.08	0.004	0.077	Light	0.01	0.000	0.009
**Evenness**							
2012				2013			
Explanatory variable	Σ*w*_i_	Model-average estimate	Unconditional SE	Explanatory variable	Σ*w*_i_	Model-average estimate	Unconditional SE
Tree mortality	0.05	0.021	0.012	Tree mortality	0.04	0.017	0.010
Soil moisture	0.03	0.019	0.012	Nutrients	0.01	-0.005	0.010
Nutrients	0.01	-0.004	0.012	Soil moisture	0.01	-0.001	0.010
Light	0.01	0.002	0.012	Light	0.01	-0.000	0.002

The most likely explanatory variables are shown in bold (based on model-average estimate being different from zero when the confidence interval excludes zero).

**Fig 2:**
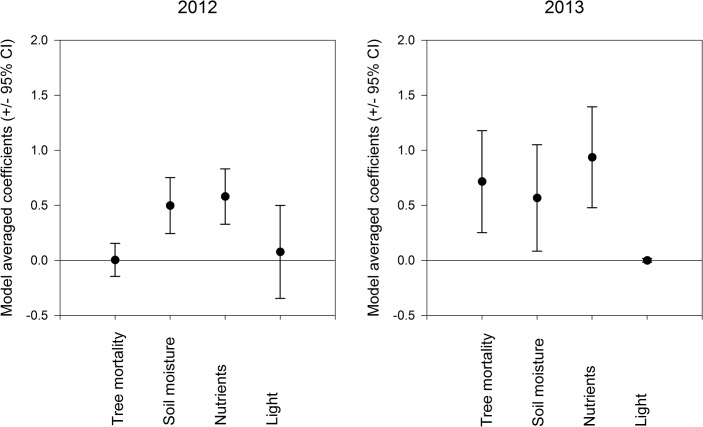
Model-averaged coefficients and 95% confidence intervals of *Dendroctonus ponderosae*-induced tree mortality, percent soil moisture, soil nutrients and light predicting understory plant community richness in 2012 and 2013.

Model selection resulted in six models for 2012 and two models for 2013 used for inference on the factors underlying richness of the understory community. Tree mortality (Σ*w*_i_ = 0.94) was found to be an important predictor of understory community richness only in 2013 (Tables 1 and 2; Fig 2; S3 Fig). Soil moisture (2012; Σ*w*_i_ = 0.56, 2013; Σ*w*_i_ = 1.00) and soil nutrients (2012; Σ*w*_i_ = 0.67, 2013; Σ*w*_i_ = 1.00) were most important in both years (Tables 1 and 2, Fig 2). Understory community richness increased with soil moisture and soil nutrients in both years, and with tree mortality only in 2013 (S3 Fig). Light availability was relatively unimportant to either understory community richness or evenness (Tables 1 and 2; Fig 2). In addition, tree mortality (2012; Σ*w*_i_ = 0.05, 2013; Σ*w*_i_ = 0.04), soil moisture (2012; Σ*w*_i_ = 0.03, 2013; Σ*w*_i_ = 0.01), and nutrient levels (2012; Σ*w*_i_ = 0.01, 2013; Σ*w*_i_ = 0.01) were not related to understory community evenness (Tables 1 and 2).

**Fig 3:**
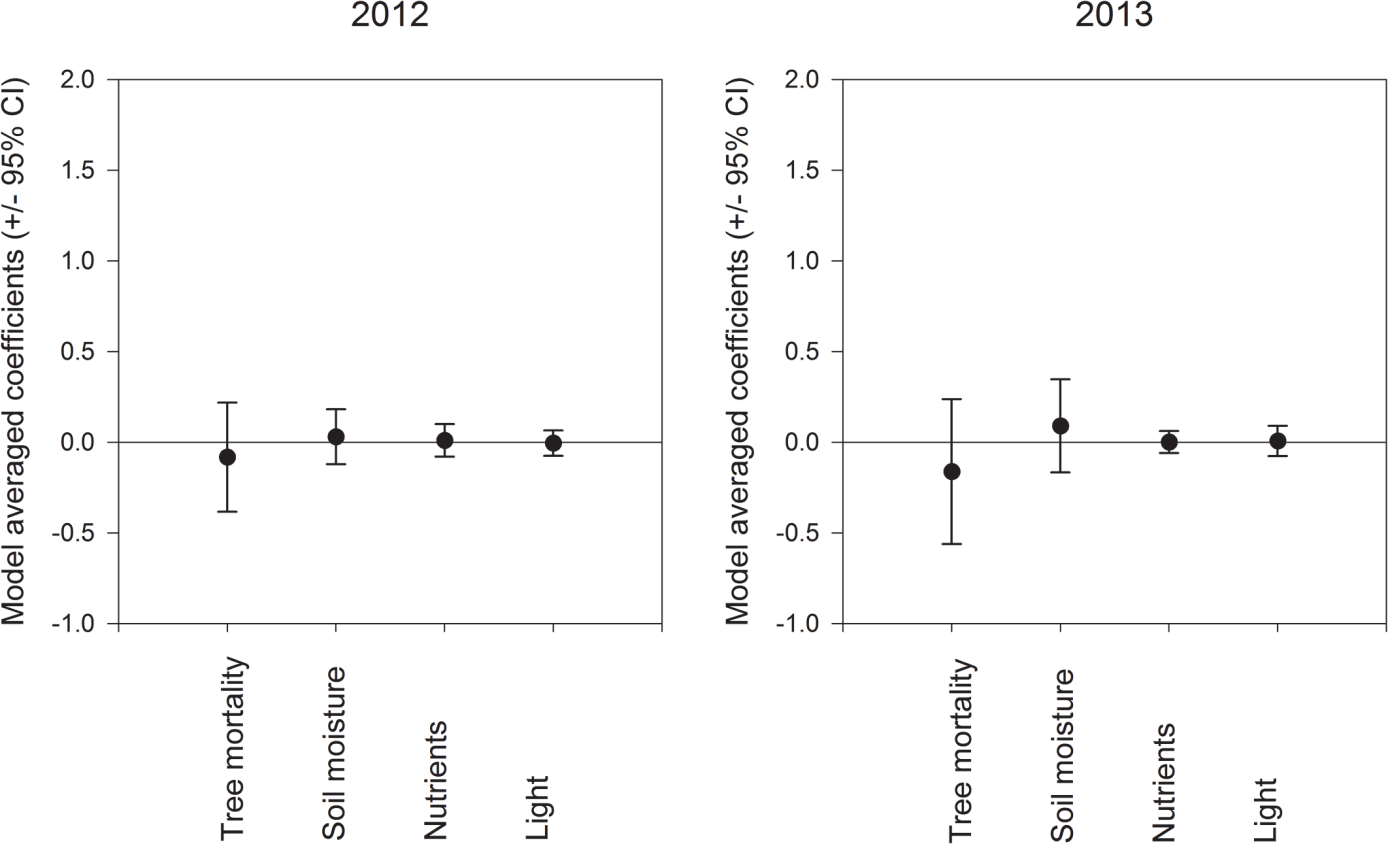
Model-averaged coefficients and 95% confidence intervals of *Dendroctonus ponderosae*-induced tree mortality, percent soil moisture, soil nutrients and light predicting evenness of understory woody perennial species in 2012 and 2013.

Underlying the effects of *D*. *ponderosae*-induced tree mortality on the understory community as a whole, were pronounced differences in responses among herbaceous and woody perennials. Model selection resulted in five models for 2012 and one model for 2013 for inference on the factors underlying richness of herbaceous perennials. Four models for both 2012 and 2013 resulted for model selection on the factors underlying richness of woody perennials. Richness of herbaceous perennials was best explained by soil nutrients in 2012 (evidence ratio = 1.00; Σ*w*_i_ = 0.71) and 2013 (evidence ratio = 1.00; Σ*w*_i_ = 1.00) (Tables 3 and 4), while tree mortality and soil moisture were found to be predictors of richness of herbaceous species in 2013, but not in 2012 (Tables 3 and 4). Richness of herbaceous perennials increased with soil nutrients in both years and with increased *D*. *ponderosae-*induced tree mortality and soil moisture levels in 2013 (S4 Fig). In contrast, richness of woody species was not strongly linked to increased tree mortality in both years (2012; Σ*w*_i_ = 0.25, 2013; Σ*w*_i_ = 0.43) (Tables 3 and 4; S4 Fig). Soil moisture, soil nutrients and light were also relatively unimportant (Σ*w*_i_ < 0.50) as predictors of the richness of woody species in either year (Tables 3 and 4) with model-average coefficients being small and showing no effect on the richness of woody species (Fig 3).

**Table 3:** Candidate models used for inference on the richness of herbaceous and woody understory response to *Dendroctonus ponderosae*-induced tree mortality, light, soil moisture, and nutrients.

**Richness of herbaceous perennials in 2012**					
	Candidate models	AIC_C_	ΔAIC_C_	*w*_i_	Evidence ratio
1	**Nutrients**	352.3	0.00	0.68	1.00
2	Soil moisture	355.9	3.63	0.11	6.18
3	Tree mortality	356.1	3.82	0.10	6.80
4	Light	356.6	4.29	0.08	8.50
5	Soil moisture, nutrients, light	358.3	5.99	0.03	22.6
**Richness of herbaceous perennials in 2013**					
	Candidate models	AIC_C_	ΔAIC_C_	*w*_i_	Evidence ratio
1	**Tree mortality, soil moisture, nutrients**	424.7	0.00	1.00	1.00
**Richness of woody perennials in 2012**					
	Candidate models	AIC_C_	ΔAIC_C_	*w*_i_	Evidence ratio
1	Null model (intercept only)	230.2	0.00	0.59	1.00
2	Tree mortality, soil moisture	232.0	1.71	0.25	2.36
3	Light	234.0	3.74	0.09	6.55
4	Nutrients	234.5	4.27	0.07	8.42
**Richness of woody perennials in 2013**					
	Candidate models	AIC_C_	ΔAIC_C_	*w*_i_	Evidence ratio
1	Tree mortality, soil moisture	356.9	0.00	0.45	1.00
2	Null model (intercept only)	357.0	0.10	0.43	1.04
3	Light	360.7	3.85	0.07	6.42
4	Soil moisture	361.0	4.08	0.06	7.50

*Notes*: The most likely models (*w*_i_>0.90; difference in evidence ratio>2.7) are shown in **bold**. AIC_C_ = Akaike’s Information Criterion corrected, ΔAIC_C_ = difference between AIC_Ci_ and AIC_C best model_, *w*_i_ = Akaike weight, and evidence ratio = *w*_j best model_ / *w*_i._

**Table 4:** Ranked relative importance of variables associated with richness of herbaceous and woody understory showing model estimates of slope and variance.

**Richness of herbaceous perennials**								
2012				2013				
Explanatory variable	Σ*w*_i_	Model-average estimate	Unconditional SE	Explanatory variable		Σ*w*_i_	Model-average estimate	Unconditional SE
**Nutrients**	0.71	0.416	0.183	**Tree mortality**		1.00	0.745	0.184
Soil moisture	0.14	0.202	0.189	**Soil moisture**		1.00	0.348	0.187
Light	0.11	0.162	0.188	**Nutrients**		1.00	0.714	0.183
Tree mortality	0.10	0.201	0.188	Light		0.01	0.175	0.201
**Richness of woody perennials**								
2012				2013				
Explanatory variable	Σ*w*_i_	Model-average estimate	Unconditional SE	Explanatory variable		Σ*w*_i_	Model-average estimate	Unconditional SE
Tree mortality	0.25	-0.082	0.154	Soil moisture		0.49	0.090	0.131
Soil moisture	0.25	0.030	0.078	Tree mortality		0.43	-0.162	0.204
Light	0.09	0.010	0.046	Light		0.07	0.007	0.043
Nutrients	0.07	-0.005	0.036	Nutrients		0.06	0.001	0.031

The most likely explanatory variables are shown in bold (based on model-average estimate being different from zero when the confidence interval excludes zero).

Model selection resulted in five models for 2012 and two models for 2013 for inference on the factors underlying evenness of herbaceous species, while model selection resulted in two models for both years on the factors underlying evenness of woody species. Tree mortality was the most important predictor of evenness in herbaceous perennials in 2013 (evidence ratio = 1.00; Σ*w*_i_ = 0.99), but not 2012 (evidence ratio = 89.0; Σ*w*_i_ = 0.01) (Tables 5 and 6). In contrast, evenness of woody perennials was not strongly linked to tree mortality across years (2012; Σ*w*_i_ = 0.02, 2013; Σ*w*_i_ = 0.21) (Tables 5 and 6), while both the evenness of herbaceous and woody perennials were unresponsive to levels of light (Σ*w*_i_ <0.10) and soil resource availability (Σ*w*_i_ <0.05) (Tables 5 and 6).

**Table 5:** Candidate models used for inference on the evenness of herbaceous and woody understory response to *Dendroctonus ponderosae*-induced tree mortality, light, soil moisture, and nutrients.

**Evenness of herbaceous perennials in 2012**					
	Candidate models	AIC_C_	ΔAIC_C_	*w*_i_	Evidence ratio
1	**Null model (intercept only)**	-96.1	0.00	0.89	1.00
2	Light	-91.0	5.02	0.07	12.7
3	Nutrients	-87.7	8.40	0.01	89.0
4	Tree mortality	-87.3	8.81	0.01	89.0
5	Soil moisture	-87.3	8.82	0.01	89.0
**Evenness of herbaceous perennials in 2013**					
	Candidate models	AIC_C_	ΔAIC_C_	*w*_i_	Evidence ratio
1	**Tree mortality**	-71.8	0.00	0.99	1.00
2	Null model (intercept only)	-61.3	10.55	0.01	99.0
**Evenness of woody perennials in 2012**					
	Candidate models	AIC_C_	ΔAIC_C_	*w*_i_	Evidence ratio
1	**Null model (intercept only)**	-31.0	0.00	0.98	1.00
2	Tree mortality	-23.4	7.64	0.02	49.0
**Evenness of woody perennials in 2013**					
	Candidate models	AIC_C_	ΔAIC_C_	*w*_i_	Evidence ratio
1	**Null model (intercept only)**	-89.2	0.00	0.78	1.00
2	Tree mortality	-86.6	2.55	0.22	3.54

*Notes*: The most likely models (*w*_i_>0.90; difference in evidence ratio>2.7) are shown in **bold**. AIC_C_ = Akaike’s Information Criterion corrected, ΔAIC_C_ = difference between AIC_Ci_ and AIC_C best model_, *w*_i_ = Akaike weight, and evidence ratio = *w*_j best model_ / *w*_i_.

**Table 6:** Ranked relative importance of variables associated with evenness of herbaceous and woody understory showing model estimates of slope and variance.

**Evenness of herbaceous perennials**								
2012				2013				
Explanatory variable	Σ*w*_i_	Model-average estimate	Unconditional SE	Explanatory variable		Σ*w*_i_	Model-average estimate	Unconditional SE
Light	0.07	-0.027	0.014	**Tree mortality**		0.99	0.070	0.015
Nutrients	0.01	0.009	0.014	Soil moisture		0.01	-0.003	0.016
Tree mortality	0.01	0.003	0.014	Nutrients		0.01	0.009	0.016
Soil moisture	0.01	0.003	0.014	Light		0.01	-0.041	0.016
**Evenness of woody perennials**								
2012				2013				
Explanatory variable	Σ*w*_i_	Model-average estimate	Unconditional SE	Explanatory variable		Σ*w*_i_	Model-average estimate	Unconditional SE
Tree mortality	0.02	-0.014	0.021	Tree mortality		0.21	0.007	0.014
Soil moisture	0.02	-0.012	0.021	Soil moisture		0.03	0.021	0.015
Nutrients	0.02	0.002	0.021	Nutrients		0.01	0.004	0.014
Light	0.02	-0.005	0.021	Light		0.01	-0.013	0.014

The most likely explanatory variables are shown in bold (based on model-average estimate being different from zero when the confidence interval excludes zero).

## Discussion

Within four years following beetle outbreak, productivity of understory plant communities increased along a gradient of beetle-induced tree mortality. Richness and evenness of herbaceous species also increased along the gradient of tree mortality while woody species richness and evenness did not. Our results reveal the complex drivers of understory diversity and productivity in which the magnitude of these shifts depended on the severity of beetle attack. Insect induced tree mortality is a dynamic process in which site conditions change as a function of tree death and time since infestation. The response of soils, for example, is not static with soil moisture and nutrient concentrations shifting over time [37]. Further, inter-annual changes in precipitation and temperature will have an effect on understory plants. In our study, understory diversity and productivity were also contingent upon current year microclimatic conditions. Below we discuss the relative importance of these findings.

### Effects of Tree Mortality and Resource Availability on Understory Plant Communities

In our study, understory species responded to immediate changes in increased soil moisture and soil nutrients with *D*. *ponderosae*-induced tree mortality; aboveground biomass nearly doubled across the attack gradient, while the diversity of herbaceous species increased with the rapid availability of soil nutrients and nearly doubled in the following year as soil moisture levels rose. Previous studies testing the effects of *D*. *ponderosae*-induced tree mortality on understory diversity and productivity have shown similar results. For instance, Kovacic et al. (1985) reported that understory biomass increased by 50% five years following attack by *D*. *ponderosae* in ponderosa pine (*Pinus ponderosa*) stands in eastern Colorado. Herbaceous species richness increased in attacked stands while increases in soil moisture increased plant biomass production. Likewise, Stone and Wolfe (1996) examined the response of understory vegetation to increased *D*. *ponderosae*-induced tree mortality in lodgepole pine stands of northern Utah. They found that understory diversity peaked at the highest levels of tree mortality, while understory biomass increased (>100 g m^-2^) exponentially in stands ranging from 14 to 95% tree mortality. However, another study of *D*. *ponderosae*-induced tree mortality in Colorado found no difference in understory vegetative cover during the first three years of post outbreak [38].

In addition to soil moisture and nutrients, light is also considered another major limiting factor in understory plant communities [29,30]. Most foliage, twigs and branches fall from attacked trees within the first eight to ten years of beetle attacks [39,40], with associated increases in light availability to the forest floor [8]. In our study, light levels were similar across the *D*. *ponderosae*-induced tree mortality gradient (Linear Mixed Model; *t* = 0.688, *P* = 0.492) and subsequently did not explain changes in understory diversity or productivity along this gradient. Across our sites, dead lodgepole pine trees remain in the overstory, many with needles, twigs and branches intact even in the most heavily attacked stands. In addition, advanced regeneration of shade-intolerant woody tree species (*A*. *balsamea*, *P*. *glauca*, and *P*. *mariana)* in the subcanopy established prior to the beetle outbreak provides shade that is partially characteristic of an intact overstory. For example, Ehrenfeld (1980) investigated understory species composition seven years after gypsy moth (*Lymantria dispar (L.))* infestation in a mature oak forest in eastern North America and found no differences between understory species in open gap and closed gap sites following disturbance. The study further suggested that the response of understory species to insect outbreak was dependent on the relative densities of the subcanopy [41].

### Mountain Pine Beetle Outbreaks versus Fire and Harvesting

In our study, herbaceous richness increased one-fold while herbaceous perennials became more evenly distributed across the gradient of tree mortality, but only after soil moisture and nutrients increased across years. Increases in understory species richness and productivity seen here are similar to observations during the first three years post-fire of forest understory vegetation in mixed conifer forests of northwestern Arizona [42] and in *P*. *ponderosa-Pseudotsuga menziesii* forests of central Colorado [43]. However, the possible mechanisms underlying each disturbance agent are quite different. Changes in resource availability and growing conditions for residual vegetation shortly after *D*. *ponderosae*-induced tree mortality is not as severe as those observed after stand-replacing fires which often eliminate most of the existing understory vegetation [29,44] allowing for increases in light availability, soil moisture, soil nutrients and favorable seedbed conditions for regenerating shade-intolerant tree species such as lodgepole pine [8,45]. In contrast, relatively slow changes in beetle-killed stands may prevent colonization by early-successional species and reduce tree seedling recruitment [8,46].

Similarly, studies on forest harvesting also find support for increases in understory richness and productivity following removal of overstory [29,47]. Increases in understory diversity in many sites are driven by increases in herbaceous species due to reduced disturbance to the organic layer and greater light availability reaching the forest floor [29,48]. Understory productivity can remain high following logging due to the presence of many residual late-successional herbaceous and woody species [49]. In our study, we found that understory diversity of herbaceous perennials increased and understory biomass nearly doubled across the gradient of *D*. *ponderosae*-induced tree mortality driven by initial increases in soil nutrients followed by increased soil moisture in the following year. Not surprisingly, richness and evenness of woody perennials did not change across the tree mortality gradient. Across our sites, many well established residual species (e.g. *Linnaea borealis*, *Vaccinium* sp.) are shade-tolerant, late-successional, and may be physiologically limited to rapidly increase growth rates rapidly in response to increased resource availability [3].

Griffin et al. (2013) reported a decrease in residual herbaceous cover and advanced regeneration following post-*D*. *ponderosae* salvage harvest in lodgepole pine forests of northeastern Wyoming. In addition, soil nitrogen availability increased following harvest. This increase in nutrients may have been due to a decrease in herbaceous cover from these older harvested stands [24]. This is contrary to our study in which understory productivity, particularly of herbaceous perennials, increased due to an increase in soil nutrients following *D*. *ponderosae*-induced tree mortality. In general, immediately following disturbance, there is potential for rapid loss of nutrients stored in soils due to decreased rates of biomass uptake [50]. However, the continued gain in understory productivity might allow the forest system to retain rather than lose nutrients following *D*. *ponderosae*-induced tree mortality.

## Conclusion

Understory vegetation showed an immediate response to *D*. *ponderosae*-induced tree mortality. The increase in abundance of herbaceous perennials may be attributed to a potential release from belowground competition of beetle-killed trees, similar to stand-replacing fires and harvesting. The decrease in belowground competition from the surrounding neighborhood could have allowed for rapid growth and a strong pulse of recruitment for some herbaceous species following disturbance. Similar observations of an increased response in advanced tree regeneration following *D*. *ponderosae*-induced tree mortality have been reported [7,51] and are worth further investigation for understory vegetative establishment and dispersal strategies. Understory diversity and productivity also increased as a result of increased soil moisture and nutrients, which were contingent upon current year growing conditions. Between years there was a lack of effect from light although there was a constant effect of soil moisture on forest understory vegetation. When soil nutrients became more abundant, soil moisture became increasingly important for continued and rapid increase in understory diversity. Further, our findings suggest that in sites with increased vegetation following *D*. *ponderosae*-induced tree mortality, tree seedling recruitment and forest recovery may be delayed. Measuring the pulse of tree seedling recruitment and possible continued increase and nutrient retention in residual understory vegetation will be important next steps toward assessing future trajectories of forest structure and composition.

## Supporting Information

S1 Fig Representative site design for sampling understory diversity and productivity following recent mountain pine beetle activity (since 2009) across eleven sites located within the Lower Foothills natural subregion of west central Alberta. (TIF)

S2 Fig Understory plant community biomass (g m^-2^) in 2012 and 2013 as a function of (A) *Dendroctonus ponderosae*-induced tree mortality in *Pinus contorta* forests, (B) percent soil moisture, and (C) soil nutrients. (PDF)

S3 Fig Understory plant community richness in 2012 and 2013 as a function of (A) *Dendroctonus ponderosae*-induced tree mortality in *Pinus contorta* forests, (B) percent soil moisture, and (C) soil nutrients. (PDF)

S4 Fig Richness of herbaceous and woody perennials as a function of *Dendroctonus ponderosae*-induced tree mortality in (A) 2012 and (B) 2013, (B) percent soil moisture in (C) 2012 and (D) 2013 and soil nutrients in (E) 2012 and (F) 2013. (PDF)

S1 Table A table of vascular plants present in sample plots along a gradient of lodgepole pine killed by mountain pine beetle. (DOCX)
