# Supplementary figures and images for: Rapid Increases in Forest Understory Diversity and Productivity following a Mountain Pine Beetle (Dendroctonus ponderosae) Outbreak in Pine Forests
Source: PLoS One. 2015 Apr 10;10(4):e0124691. doi: 10.1371/journal.pone.0124691 (PMC4393282; doi:10.1371/journal.pone.0124691)

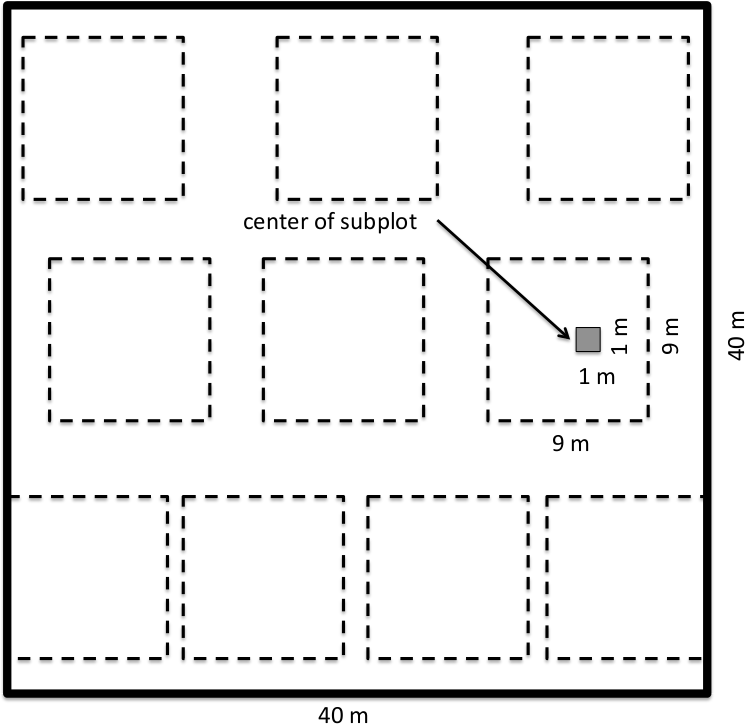

Supplement: S1 Fig — (TIF) [file pone.0124691.s001.tif]

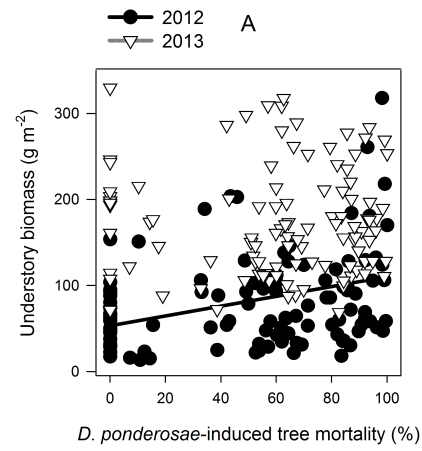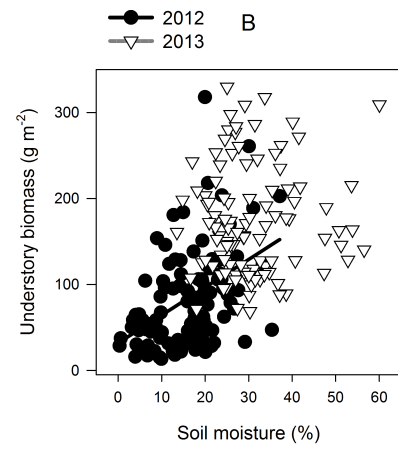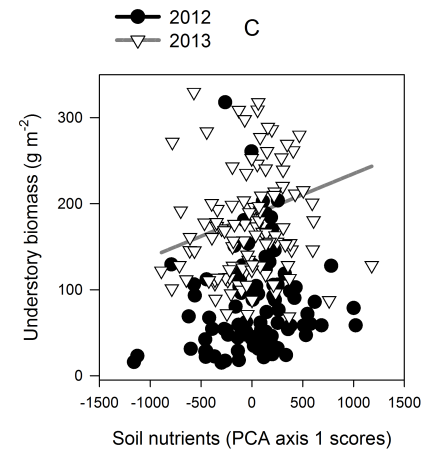

Supplement: S2 Fig — (PDF) [file pone.0124691.s002.pdf]

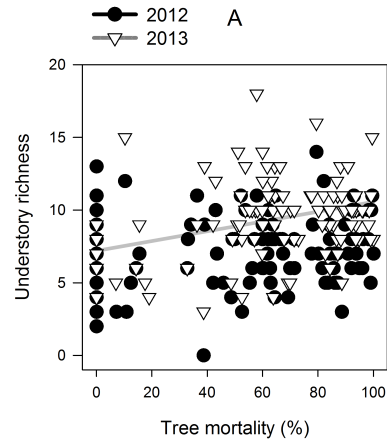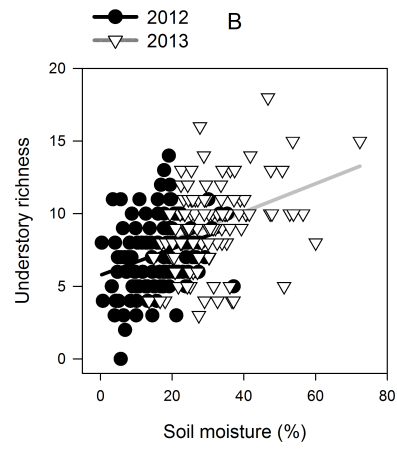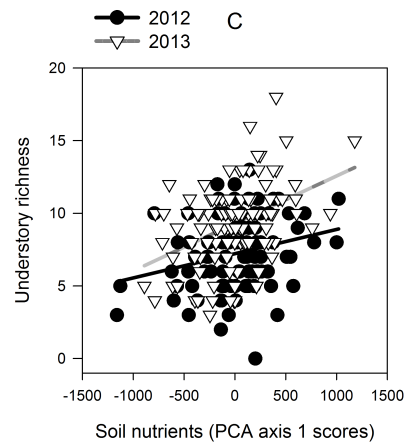

Supplement: S3 Fig — (PDF) [file pone.0124691.s003.pdf]

A) 2012

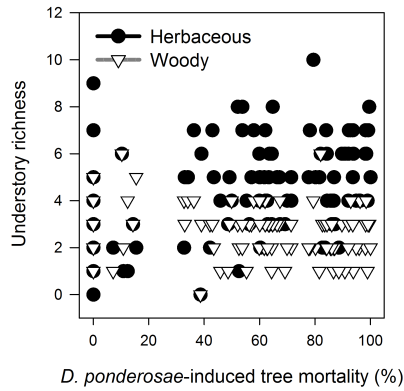

B) 2013

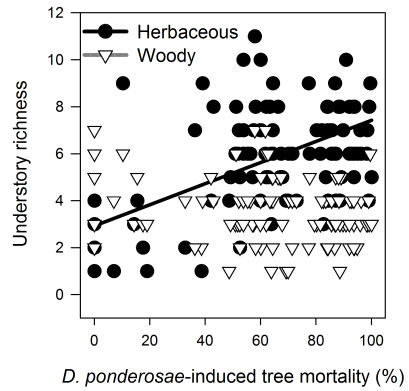

C) 2012

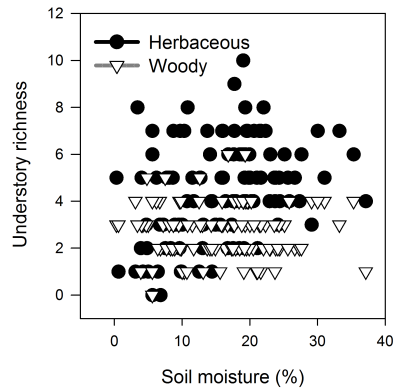

D) 2013

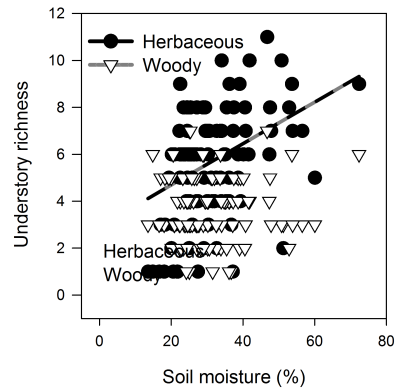

E) 2012

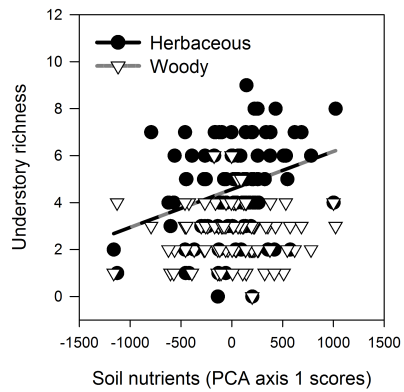

F) 2013

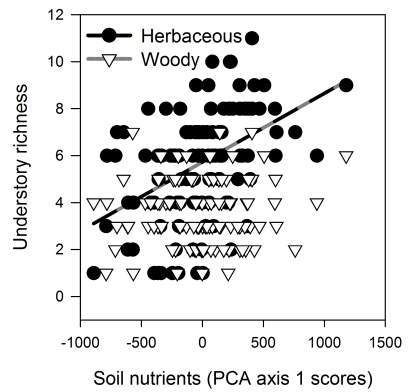

Supplement: S4 Fig — (PDF) [file pone.0124691.s004.pdf]
