# Supplementary material for: Rapid Increases in Forest Understory Diversity and Productivity following a Mountain Pine Beetle (Dendroctonus ponderosae) Outbreak in Pine Forests
Source: PLoS One. 2015 Apr 10;10(4):e0124691. doi: 10.1371/journal.pone.0124691 (PMC4393282; doi:10.1371/journal.pone.0124691)
Supplement: S1 Table — (DOCX) [file pone.0124691.s005.docx]

S1 Table. A table of vascular plants present in sample plots along a gradient of lodgepole pine killed by mountain pine beetle.

| Species | Family | Functional group |
| --- | --- | --- |
| *Actaea rubra* | Ranunculaceae | Herbaceous |
| *Amelanchier alnifolia* | Rosaceae | Woody |
| *Aralia nudicaulis* | Araliaceae | Herbaceous |
| *Arnica cordifolia* | Asteraceae | Herbaceous |
| *Aster ciliolatus* | Asteraceae | Herbaceous |
| *Athyrium filix-femina* | Dryopteridaceae | Herbaceous |
| *Cornus canadensis* | Cornaceae | Herbaceous |
| *Disporum trachycarpum* | Liliaceae | Herbaceous |
| *Epilobium angustifolium* | Onagraceae | Herbaceous |
| *Equisetum pratense* | Equisetaceae | Herbaceous |
| *Eurybia conspicua* | Asteraceae | Herbaceous |
| *Galium triflorum* | Rubiaceae | Herbaceous |
| *Goodyera repens* | Orchidaceae | Herbaceous |
| *Gymnocarpium dryopteris* | Dryopteridaceae | Herbaceous |
| *Lathyrus ochroleucus* | Fabaceae | Herbaceous |
| *Ledum groenlandicum* | Ericaceae | Woody |
| *Linnaea borealis* | Caprifoliaceae | Woody |
| *Listera cordata* | Orchidaceae | Herbaceous |
| *Lonicera involucrata* | Caprifoliaceae | Woody |
| *Lycopodium annotinum* | Lycopodiaceae | Herbaceous |
| *Lycopodium complanatum* | Lycopodiaceae | Herbaceous |
| *Maianthemum candense* | Liliaceae | Herbaceous |
| *Mertensia paniculata* | Boraginaceae | Herbaceous |
| *Mitella nuda* | Saxifragaceae | Herbaceous |
| *Oplopanax horridus* | Araliaceae | Woody |
| *Orthilia secunda* | Pyrolaceae | Woody |
| *Petasites palmatus* | Asteraceae | Herbaceous |
| *Pyrola asarifolia* | Pyrolaceae | Woody |
| *Pyrola chlorantha* | Pyrolaceae | Woody |
| *Pyrola uniflora* | Pyrolaceae | Herbaceous |
| *Ribes lacustre* | Grossulariaceae | Woody |
| *Rosa acicularis* | Rosaceae | Woody |
| *Rubus parviflorus* | Rosaceae | Woody |
| *Rubus pedatus* | Rosaceae | Herbaceous |
| *Rubus pubescens* | Rosaceae | Herbaceous |
| *Smilacina racemosa* | Liliaceae | Herbaceous |
| *Spiraea betulifolia* | Rosaceae | Woody |
| *Streptopus amplexifolius* | Liliaceae | Herbaceous |
| *Tiarella trifoliata* | Saxifragaceae | Herbaceous |
| *Trifolium pratense* | Fabaceae | Herbaceous |
| *Vaccinium caespitosum* | Ericaceae | Woody |
| *Vaccinium myrtilloides* | Ericaceae | Woody |
| *Vaccinium vitis-idaea* | Ericaceae | Woody |
| *Viburnum edule* | Caprifoliaceae | Woody |
| *Viola renifolia* | Violaceae | Herbaceous |
